# Subglottic Hemangioma Treated With Propranolol

**Published:** 2014-01-15

**Authors:** Scott A. Hardison, Kelley M. Dodson, Jennifer L. Rhodes

**Affiliations:** ^a^Department of Otolaryngology—Head and Neck Surgery; ^b^Department of Surgery, Division of Plastic and Reconstructive Surgery, Virginia Commonwealth University/Medical College of Virginia, Richmond, Va

**Keywords:** propranolol, subglottic hemangioma, hemangioma, airway, stridor

## DESCRIPTION

A 3-month-old girl child was admitted to the pediatrics service with a 1-month history of respiratory distress and stridor, which had acutely worsened over the previous day and led to a brief episode of apnea. The stridor was biphasic in nature with a more pronounced inspiratory component and was exacerbated by agitation and supine positioning. Initial bedside fiberoptic examination by otolaryngology did not reveal any clear structural abnormalities, but it was unable to assess beyond the vocal cords. Flexible bronchoscopy on hospital day 4, however, revealed severe narrowing of the subglottis from a left-sided lesion. She was transferred to the pediatric intensive care unit for closer monitoring and a subsequent computed tomography angiogram (CTA) of the neck revealed a large subglottic hemangioma.

## QUESTIONS

1. **Discuss the pathogenesis and epidemiology of subglottic hemangiomas. What clinical clues can suggest the presence of a subglottic hemangioma?**
2. **What treatment options exist for this condition?**
3. **What pretreatment workup should be performed before initiating propranolol therapy?**
4. **What adverse effects may be seen with propranolol therapy?**

## DISCUSSION

A subglottic hemangioma (SGH) is a benign tumor of infancy that can cause severe obstruction of the airway. Overall, infantile hemangiomas affect 4% to 5% of the pediatric population, making them the most common type of head and neck tumor in children.^1^ In the pediatric airway, however, these growths account for only 1.5% of congenital abnormalities.^2^ This rarity, coupled with a mortality rate of close to 50% when left untreated,^3^ means that an SGH poses a great challenge to clinicians evaluating children for respiratory distress. From an epidemiologic standpoint, SGHs occur most often in female patients (2:1) and have been linked to low birth weight and prematurity.^4^ SGHs, like other infantile hemangiomas, follow a well-described pattern of proliferation beginning in the first 1 to 3 months of life, followed by involution by about 1 year of age.^5^^,^^6^ It is during the early proliferative phase that patients become symptomatic, developing characteristic biphasic stridor, which may progress to respiratory distress. In this early stage, an SGH is often mistaken for a more common condition such as croup, even presenting with a similar “barking” cough in some instances.^6^ In patients with SGH, however, conservative interventions such as racemic epinephrine and steroids are only transiently helpful.^6^ Thus, patients with “recurrent croup” are prime candidates for further evaluation for SGH, especially when their episodes of respiratory distress are worsening and are not associated with fever or rhinorrhea.^6^ Though SGHs often occur in isolation, the presence of cutaneous hemangiomas, especially in the “beard” distribution (preauricular areas, chin, anterior neck, and lower lip), may also suggest the presence of an SGH in children with respiratory distress.^7^ In the case mentioned earlier, the patient's more prominent inspiratory component to her stridor led to initial misdiagnosis as laryngomalacia by her primary care physician.

Over the past few decades, treatments for SGH have been evolving. These treatment modalities include systemic corticosteroids,^8^ intralesional steroids,^9^ carbon dioxide laser,^8^ tracheostomy, and open surgical resection.^10^^-^12 Systemic corticosteroids were long seen as the initial treatment of choice, as 25% of lesions respond to this method, but this may expose the child to the well-described negative effects of systemic steroid use.^8^ Intralesional steroids have shown 82% effectiveness in one study but required a mean of 6 procedures and 37 days of intubation to achieve success.^9^ Likewise, laser treatment has produced success rates as high as 89% but with a high rate of complications like subglottic stenosis, limiting their use to smaller, noncircumferential lesions.^8^ Tracheostomy may also be employed, as SGHs will spontaneously involute after about 1 year of life, but this may lead to tracheal stenosis or tracheocutaneous fistulae following decannulation. Open surgical excision is often seen as the criterion standard for definitive management of SGH, achieving success rates as high as 94% with a relatively low complication rate using modern reconstructive techniques.^8^^,^^10^^,^^11^ In 2008, however, a landmark paper in the *New England Journal of Medicine* observed that a child with a large infantile hemangioma who was being treated with propranolol for an unrelated hypertrophic cardiomyopathy exhibited rapid regression of the hemangioma within days of initiating propranolol.^12^ When propranolol was given to 10 additional patients, they similarly exhibited remarkable results within 24 hours of initiating therapy.^12^ Since that time, propranolol has become the new first-line treatment for infantile hemangiomas, including those of the subglottis, producing excellent results in a number of case reports and retrospective series.^5^^,^^13^^-^16

The Great Ormond Street Hospital recently published a protocol for the use of propranolol in the treatment of isolated SGH, which outlines appropriate pretreatment workup and treatment dosing.^17^ Key among these preparations are electrocardiography and echocardiography, helping to avoid exacerbation of preexisting cardiac problems. A baseline pulse, blood pressure, and blood glucose should also be assessed. Treatment commonly involves initiating therapy at 1 mg/kg/day for 1 week, then advancing to 2 to 3 mg/kg/day as tolerated.^5^^,^^13^^-^17 Vital signs should be monitored closely at the initiation of therapy and whenever the dose is increased.^17^ Dosing must be adjusted over the subsequent months to adjust for the growth of the child.^17^ The proposed Great Ormond Street guidelines advocate treatment for 12 months to cover the natural period of proliferation, then tapering of the propranolol dose over 4 weeks.^17^ These children should be monitored closely after treatment for recurrence.

Adverse effects of propranolol are rare but may include bradycardia, hypotension, and hypoglycemia.^17^ Other potential adverse effects include bronchospasm, fatigue, nightmares, heart failure, and peripheral vasoconstriction.^17^ The benefits of propranolol therapy, however, are generally considered to outweigh its risks, especially as compared to surgical morbidity and the systemic effects of steroids.

Following diagnosis, our 3-month-old patient was started on prednisolone and underwent an echocardiogram, electrocardiogram, and blood glucose check. After her workup was complete, she received an initial dose of propranolol at 0.5 mg/kg, which was advanced to 1 mg/kg after no adverse effects were noted at 4 hours. She was continued at 1 mg/kg every 8 hours (total 3 mg/kg/day) for the remainder of her hospitalization with now ill effects noted. Significant improvements in stridor and respiratory effort were noted within 48 hours, with discharge taking place less only a week after initiating propranolol therapy. Repeat bronchoscopy after 3 weeks of treatment revealed a 75% reduction in the size of the lesion. Her dose has been continuously adjusted for increases in weight and at a recent 10-month follow-up visit she demonstrated no evidence of recurrence.

## Figures and Tables

**Figure 1:**
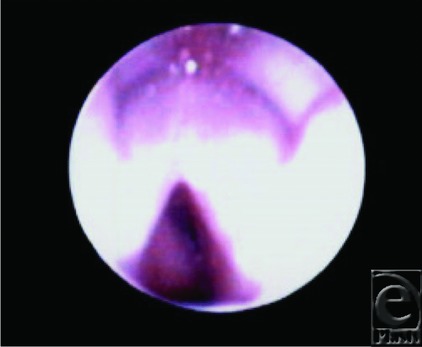
Pretreatment tracheobronchoscopy.

**Figure 2:**
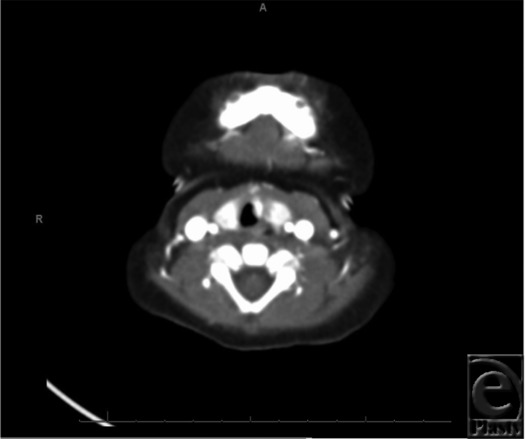
Pretreatment CTA of the neck.

**Figure 3:**
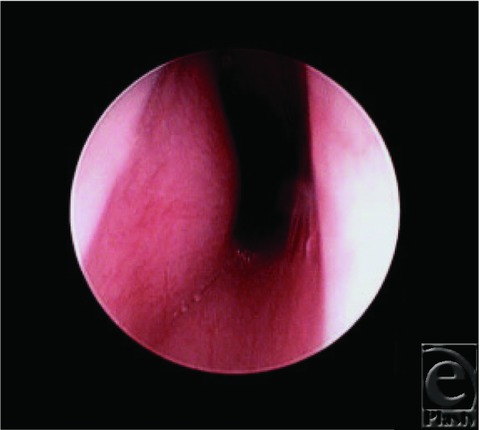
Tracheobronchoscopy 3 weeks posttreatment.
